# Breast cancer detection using enhanced IRI-numerical engine and inverse heat transfer modeling: model description and clinical validation

**DOI:** 10.1038/s41598-024-53856-w

**Published:** 2024-02-09

**Authors:** Carlos Gutierrez, Alyssa Owens, Lori Medeiros, Donnette Dabydeen, Nithya Sritharan, Pradyumna Phatak, Satish G. Kandlikar

**Affiliations:** 1 Rochester Institute of Technology, Rochester, USA; 2 Rochester Regional Health, Rochester, USA

**Keywords:** Mechanical engineering, Computational science, Medical imaging, Breast cancer

## Abstract

Effective treatment of breast cancer relies heavily on early detection. Routine annual mammography is a widely accepted screening technique that has resulted in significantly improving the survival rate. However, it suffers from low sensitivity resulting in high false positives from screening. To overcome this problem, adjunctive technologies such as ultrasound are employed on about 10% of women recalled for additional screening following mammography. These adjunctive techniques still result in a significant number of women, about 1.6%, who undergo biopsy while only 0.4% of women screened have cancers. The main reason for missing cancers during mammography screening arises from the masking effect of dense breast tissue. The presence of a tumor results in the alteration of temperature field in the breast, which is not influenced by the tissue density. In the present paper, the IRI-Numerical Engine is presented as an adjunct for detecting cancer from the surface temperature data. It uses a computerized inverse heat transfer approach based on Pennes’s bioheat transfer equations. Validation of this enhanced algorithm is conducted on twenty-three biopsy-proven breast cancer patients after obtaining informed consent under IRB protocol. The algorithm correctly predicted the size and location of cancerous tumors in twenty-four breasts, while twenty-two contralateral breasts were also correctly predicted to have no cancer (one woman had bilateral breast cancer). The tumors are seen as highly perfused and metabolically active heat sources that alter the surface temperatures that are used in heat transfer modeling. Furthermore, the results from this study with twenty-four biopsy-proven cancer cases indicate that the detection of breast cancer is not affected by breast density. This study indicates the potential of the IRI-Numerical Engine as an effective adjunct to mammography. A large scale clinical study in a statistically significant sample size is needed before integrating this approach in the current protocol.

## Introduction

Breast cancer is one of the most common cancers among women and the second leading cause of death in women. In 2022, it was estimated that about 287,850 women were diagnosed with invasive breast cancer and 43,250 women died from breast cancer, in the United States alone^1^. Due to advancements in treatment and early detection, the death rate of breast cancer in the US had declined by 42% from 1989 to 2019^1^. Early detection of breast cancer is associated with improved chances of survival and options for curative treatment^2^. Mammography has been used since the 1970s for breast cancer screening in asymptomatic women. Screening mammography in women aged 40–74 is associated with a relative reduction in breast cancer mortality of 15–20%^3^. Breast density is an important consideration in breast cancer screening. The four categories of breast density are fatty, scattered fibroglandular, heterogeneously dense, and extremely dense tissue. Heterogeneously dense and extremely dense breasts are considered “dense breasts,” found in > 40% of women who are at a higher risk of developing cancer^4^. The sensitivity of mammography is estimated at 77–95%, and the specificity is estimated at 92–97% for a randomized single exam but was as low as 76% in cumulative specificity over a 10-year period^2,5,6^. However, mammography is suboptimal in dense breasts, mainly due to masking effects from dense tissue, resulting in ~ 38% tumors to be missed or misdiagnosed^6^.

Other imaging modalities that have been utilized for breast cancer screening are ultrasound and magnetic resonance imaging (MRI). Ultrasound is the most common adjunct to mammography that has improved sensitivity to 97.3% and specificity to 76.1% when combined with mammography^2^. Ultrasound is the preferred method for screening dense breast tissue, but the low specificity from high false positive rates makes it only as reliable as utilizing only mammography. For high-risk patients, MRI is a preferred adjunct to mammography with a sensitivity of 94% and specificity of 77% when combined with mammography^2^. MRI is also great in imaging dense breast tissue, but it is typically reserved for high-risk breast cancer patients due to very high cost. Although these modalities help improve the sensitivity, the value of the specificity remains much lower, showing the need for better technology. Development of computer-aided diagnostic software using artificial intelligence (AI) with these imaging modalities has shown great promise in further advancing current screening^7,8^. In literature, some alternative systems are being proposed for breast imaging and cancer detection such as the iBreast^9^ and the wearable ultrasound breast patch^10^. The iBreast^9^ is a device invented to measure the elasticity of the breast tissue and compare it with a database containing measurements for healthy breast tissue to detect the presence of cancer. The wearable ultrasound breast patch is an ultrasound-based wearable device, which is the first of its kind, and it scans breast tissue for small cysts utilizing similar concepts as the iBreast. These devices showed the potential of utilizing measured quantitative physical data to infer the presence of breast tumors or lesions. However, large-scale clinical testing is needed to further validate these devices.

Three major concerns facing women with annual mammography are: (a) missed tumors in dense breasts, (b) anxiety related to recall and follow up testing with an additional mammogram, ultrasound/biopsy or MRI when the initial mammogram cannot provide clear assessment, and (c) radiation exposure and discomfort due to compression. Similarly, major concerns facing a radiologist are: a) tumors masked by dense breast tissue (missed cancer), and (b) constraints balancing recall rates—a higher recall rate, which increases healthcare cost and further increases radiation exposure, detects more cancers but leads to larger number of false positives and unnecessary testing in healthy women, and a lower recall rate that lowers cancer detection rate. Recall rate is an important consideration in mammography. Grabler et al.^11^ studied its effect on cancer detectability in different groups of recall rates from 7 to 20%. A higher recall rate can help in detecting early-stage cancers, which would be missed with low recall rates. However, a higher recall rate also results in a significantly higher number of additional tests, including biopsies. Grabler et al.^11^ suggest using the tumor size as a criterion in deciding the recall rate. This clearly indicates that the low specificity of mammography needs to be improved by a successful adjunctive modality since it leads to such a large number of recalls (10% or higher of screenings).

Infrared (IR) thermography is a technique that is used to find temperature differences, patterns, or distributions on the surface of an object. These temperatures are measured using an IR camera, which captures IR radiation emitted from the surface of the object and transmits it as a thermal image, or IR image. Thermal imaging techniques, which relied on thermal gradients and hot spots in the skin surface temperature profiles, have been used in the past with variable but generally poor results leading to their bad repute among radiologists. Current modern IR cameras have become relatively inexpensive (< $20–30 K) and have high temperature sensitivity (~ 20 mK). Kandlikar et al.^12^ presented a detailed review of breast thermography for detecting breast cancer. The need for a validated IR technology based on a rigorous analytical framework was indicated. Infrared thermography, also known as infrared imaging (IRI), has no harmful radiation and is a non-invasive technique that is unaffected by tissue density. Recent clinical trials using breast thermography have employed dynamic IRI^12–16^, which relies on dynamic surface temperature response on the breast surface from external airflow. Gonzalez-Hernandez et al.^15^ discussed the current status and recent advances in the field of dynamic breast IRI. Patient discomfort due to uneven airflow around the breast during dynamic IRI are some of the major issues.

Steady-state IRI mainly relies on obtaining gradient and mean temperatures on 2D images of the breast surface and developing empirical and library-based tools to determine the presence of cancer^15,17–21^. Current IRI approach lacks scientific rigor and has not been validated in a statistically significant pool of patients in clinical trials. Infrared imaging is not proposed as an alternative to mammography but has been cleared by the FDA as an adjunct to mammography, similar to ultrasound^22^. The advancements of IRI technology and acceptance by the FDA as an adjunctive medical tool has inspired others to investigate utilizing IRI in various medical applications. Bhowmik et al^23^ developed a new portable blood perfusion imager that has shown the ability of utilizing IRI to screen oral cancer. The work utilized traditional AI-based methods with thermal analysis of cancer vascularization to classify oral cancer and precancer. This shows the capability of IRI for cancer screening when utilized with thermal-based methods. A recent review by Mashekova et al^21^ has shown the various techniques utilized with IRI to detect breast cancer through a thermal perspective.

In the 1970s and 1980s, Gautherie^24^ investigated thermopathological, or thermal-based pathology, correlations of breast tumors using IRI. He conducted temperature and effective thermal conductivity measurements through a probe as well as by taking IR images of the breast surface. In his study, the IRI temperatures of a breast with breast cancer were higher than that of the contralateral healthy breast. He observed that the measured internal temperature for a breast with a tumor is much higher than that of a healthy breast. An increase in the measured effective thermal conductivity in the region with increased surface temperature was also observed. Gautherie^24^ correlated these findings to increased blood supply in the tumor region, or hypervascularization, and due to an increase in metabolic activity. This study showed that further research was needed to understand this thermal phenomenon caused by breast tumors and captured by IRI.

Thermal modeling of breast cancer has played a major role in further developing and understanding IRI research with the use of bioheat transfer modeling. Pennes’s bioheat equation^25^, given below, has played a major role in bioheat transfer modeling and forms the basis for many complex models:

1 $$\rho_{t} c_{t} \left( {\frac{{\partial T_{t} }}{\partial t}} \right) = \nabla \cdot \left( {k_{t} \nabla T_{t} } \right) + \rho_{b} c_{b} \omega_{b} \left( {T_{a} - T_{t} } \right) + q_{m}$$

where $\rho$ is density, $c$ is the specific heat, $T$ is temperature, $t$ is time, $k$ is the thermal conductivity, $\omega_{b}$ is the blood perfusion rate, and $q_{m}$ is the metabolic heat generation rate, while the subscripts $t$, $b$ and $a$ stand for tissue, blood, and artery, respectively. There have been variations to Pennes’s model, such as the generic bioheat transfer model^26^, and the coupled continuum-discrete bioheat model^27^. There have been many geometric models, with specific thermal properties, boundary conditions, and bioheat models that were utilized to obtain simulated surface temperatures of a breast with cancer^15,16,21,28,29^.

A recent review by Mashekova et al.^21^ covered techniques such as AI-based methods, bioheat transfer modeling, and inverse algorithms that utilized IRI for breast cancer detection. According to the authors, three main geometric models that have been studied extensively in bioheat thermal simulations of breast cancer are: (i) rectangular geometric models, (ii) hemispherical, or idealized spherical, geometric models, and (iii) realistic breast models. These geometries aided in fundamental studies to understand the significance of tumor depth and correlations of tumor diameter with heat generation based on the work by Gautherie^24^. Furthermore, these studies aided in identifying the appropriate boundary conditions for modeling and understanding the importance of tissue layers in the model. From these studies, it is now known that tumors of sizes 1.0–3.6 cm that are as deep as 1.8 cm within the breast will have a noticeable surface temperature variation. With the increased thermal sensitivity of the infrared cameras, even smaller temperature differences can be noticed to see thermal variations from deeper tumors. Also, these studies showed that multiple tissue layers did not have a significant effect in the temperature distribution, allowing for a simplified model. Some of these studies were able to predict surface temperatures that were in agreement with the experimental studies conducted by Gautherie^24^.

Various thermal properties and boundary conditions, including the values for heat generation, heat transfer coefficient, thermal conductivity, and blood perfusion rate, were taken from known experiments and studies^21,28–30^. These studies have utilized bioheat transfer modeling to uncover the appropriate conditions to compare thermal surface temperature simulations with IR images. Inverse heat transfer studies, such as the work by Figueiredo et al.^31,32^ have shown the ability to detect breast cancer in a 2D realistic model from the surface temperatures. These models utilized the fact that a metabolically active tumor acts as a heat source and the blood perfusion rate is higher for tumors in comparison to healthy tissue, as reported by Gautherie^24^. Camilleri et al.^28^ conducted a review of thermal and physiological properties of breast tissue based on some of these previous studies. These studies laid the foundation of utilizing thermal modeling and IRI to understand the role of heat transfer in breast cancer. However, very few studies utilized patient IR data from IRI with patient-specific 3D geometries, such as the work from Gonzalez-Hernandez et al.^33,34^. The inverse heat transfer approach has shown to be very effective in obtaining thermal properties such as boundary conditions^35^, heat sources^36^, thermal conductivity^37^ and other parameters^38–40^.

The present work uses patient IRI data, patient-specific breast geometries, and inverse heat transfer modeling for detecting the presence and absence of breast cancer. Gonzalez-Hernandez et al.^33^ developed a method for generating patient-specific breast geometries from MRI for bioheat thermal simulations of breast cancer. Gonzalez-Hernandez et al.^34^ then utilized these methods with patient IRI data in an inverse heat transfer approach to detect breast tumors. Gonzalez-Hernandez et al.^34^ and Recinella et al.^41^ were able to validate this method on seven biopsy-proven breast cancer patients recruited in a clinical study at Rochester General Hospital (RGH). The results showed great promise in detecting the presence and absence of breast tumors from IR images regardless of breast density. It should be noted that the MRI data was used only for obtaining the breast outline; it was not used in the inverse heat transfer modeling algorithm. However, more efficient registration process and algorithm were needed to process a larger data set, and further validation of the approach was needed on the remaining patient cohort. An enhanced detection algorithm has been developed in this work that utilizes these methods in a packaged algorithm called the IRI-Numerical Engine. Enhancements were conducted to extend this study to a larger cohort of patient data. The aim of the current work is as follows:

1. Validate the IRI-Numerical Engine using patient-specific 3D geometries and surface temperatures from patient IRI data.
2. Evaluate the efficacy of the IRI-Numerical Engine in a sample size of twenty-three patients.
3. Show the capability of the IRI-Numerical Engine to detect breast cancer regardless of breast density or cancer type.
4. Further advance the research of IRI as an adjunct to mammography through the IRI-Numerical Engine.

This work is a continuation and enhancement of the work conducted by Gonzalez-Hernandez et al.^34^ and Recinella et al.^41^ using additional patient data for validation. The algorithm was improved to eliminate the time-consuming manual registration process in the previous work. The uniqueness of the present work is the algorithm’s efficiency in correctly detecting the presence and absence of breast cancer from the surface temperatures from patient IRI data using patient-specific digital breast models.

## Results

The breast density, tumor grade and cancer type for twenty-three patients collected at RGH are shown in Table 1. The breast density types observed in the patient cohort are predominantly fatty (PF), scattered fibroglandular (SF), heterogeneously dense (HD), and extremely dense (ED). Table 1 shows that five of the patients have dense breast tissue types and the remaining have fatty breast tissue types. The tumor grade is an indicator on how abnormal cancer cells look, grow, and spread with values of X (undetermined grade), 1 (low grade), 2 (intermediate grade), 3 (high grade), and 4 (high grade)^42^. In the patient cohort, only one patient had undetermined grade while the rest had tumor grades from 1 to 3. The cancer types observed in these patients are ductal carcinoma in situ (DCIS), invasive ductal carcinoma (IDC), invasive lobular carcinoma (ILC), lobular carcinoma in situ (LCIS), and atypical ductal hyperplasia (ADH). One patient was found to have bilateral breast cancer (Patient 4), where the right breast was diagnosed with IDC and the left breast was diagnosed with ILC.

**Table 1:** Patient data of all twenty-three patients collected for clinical study at Rochester General Hospital including the patient’s age, breast with tumor (BWT), breast density, tumor grade, and cancer type.

Patient	Age	BWT	Breast density	Tumor grade	Cancer type
1	60	R	HD	2	DCIS
2	70	R	SF	2	IDC
3	71	R	PF	1	IDC
4	68	R	SF	3	IDC
4	68	L	SF	1	ILC
5	51	R	SF	2	IDC
6	67	L	SF	1	IDC
7	67	L	SF	1	IDC
8	62	L	PF	3	ILC
9	46	R	HD	2	IDC
10	48	R	SF	1	IDC
11	64	R	PF	1	IDC
12	68	L	HD	1	ADH
13	68	L	SF	3	IDC
14	70	R	SF	3	IDC
15	42	R	HD	3	IDC
16	49	R	SF	3	IDC
17	70	L	SF	2	ILC
18	67	L	ED	X	LCIS
19	72	R	SF	2	IDC
20	72	L	SF	3	IDC
21	64	L	SF	2	IDC
22	63	L	SF	2	IDC
23	57	L	SF	2	IDC

The letters R and L represent the right and left breast, respectively. Patient 4 has bilateral breast cancer and appears twice in the table, but with each breast having distinct tumor grades and cancer types. Patient 18 is the only one with an undetermined tumor grade that is represented by X. The breast density types are predominantly fatty (PF), scattered fibroglandular (SF), heterogeneously dense (HD), and extremely dense (ED). The cancer types are ductal carcinoma in situ (DCIS), invasive ductal carcinoma (IDC), invasive lobular carcinoma (ILC), lobular carcinoma in situ (LCIS), and atypical ductal hyperplasia (ADH).

**Figure 1:**
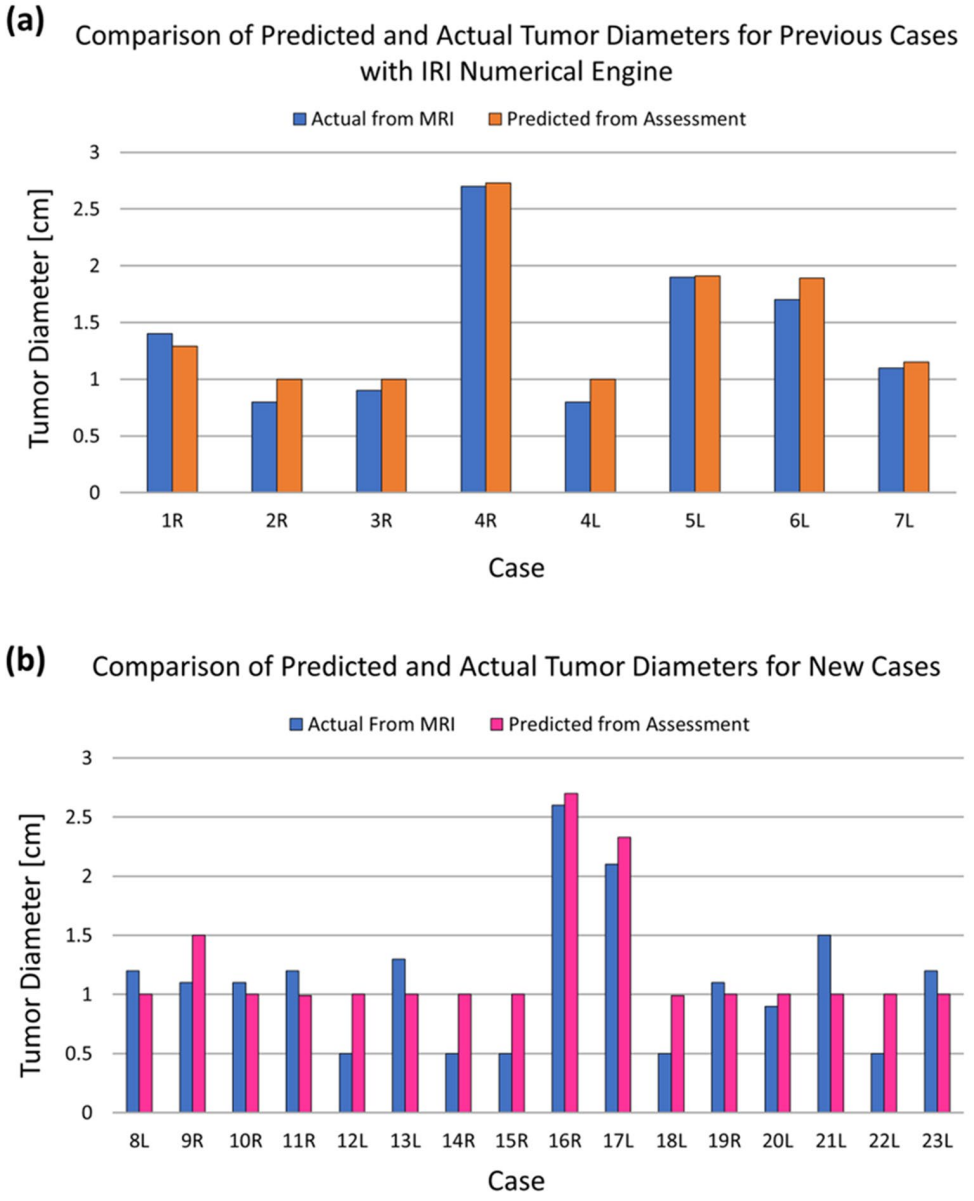
Validation results of the IRI-Numerical Engine for tumor size utilized on the (**a**) previous seven cases and (**b**) additional sixteen new cases comparing with actual size from MRI. Not shown in the figure are the results for healthy breast, as no tumors were detected for contralateral healthy breast.

The first seven patients were studied previously by Gonzalez-Hernandez et al.^34^ to validate the use of an inverse heat transfer algorithm that was developed for breast cancer detection using IR images. In this study, these seven patient cases were utilized for initial validation of the new enhanced IRI-Numerical Engine, and subsequently tested on the additional sixteen patients. Figure 1 shows the results of detection from the IRI-Numerical Engine on the previous patient cases (Fig. 1a) and on the new patient cases (Fig. 1b). The results shown in Fig. 1a validates that the IRI-Numerical Engine is able to obtain similar results presented by Gonzalez-Hernandez et al.^34^. As Fig. 1 shows, the algorithm was able to detect the presence of tumors for all patient cases. The absence of a tumor in the contralateral healthy breast, except for Patient 4, was also correctly predicted for all cases. Patient 4 was the only patient case with bilateral breast cancer, giving a total of twenty-four breast cases with cancer and twenty-two breast cases without cancer. The presence and absence of the tumor was detected regardless of breast density, tumor grade, or cancer type. The algorithm determined the absence of a tumor by moving the tumor, during iterations, outside of the breast or at the chest wall boundary as no heat generating tumor was detected in the breast domain. The algorithm did not utilize any prior knowledge of the tumor size or location from MRI or other medical reports. Gonzalez-Hernandez et al.^34^ showed that the final results for tumor characteristics, if present, are independent of the initial guesses. In all cases, the initial guess for tumor location was at the center of the breast irrespective of the IR images. Thus, the IRI-Numerical Engine was able to correctly predict the presence of twenty-four breasts with cancer and correctly predict the absence of tumors in twenty-two contralateral breasts without tumors.

Table 2 shows the predicted tumor diameter from the algorithm, the actual tumor diameter obtained from MRI, as well as the error and absolute error in tumor size prediction for each patient. The predicted tumor diameter values range from 10 mm to 27.3 mm while the actual tumor diameter values range from 5 to 27 mm. The minimum diameter of a tumor is set as 10 mm as no metabolic rate data is available below 10 mm as provided by Gautherie^24^. Further research is warranted in this area. The mean and median errors in tumor size prediction were 1 mm for both while the minimum and maximum errors were 5 mm and − 5 mm, respectively. The negative value in the errors indicate that the algorithm predicted a smaller tumor diameter value than the actual tumor diameter. The maximum, mean, and median absolute errors in tumor size prediction were 5 mm, 2.4 mm, and 2 mm, respectively. The predicted tumor diameters provided by the algorithm are associated with an equivalent tumor heat source to match the surface temperatures from the IR images. The IRI-Numerical Engine was applied to twenty-four breast cases with cancer and twenty-two breast cases without cancer. This gives a total of forty-six breast cases that were analyzed. The IRI-Numerical Engine took an average of 2.5 h for each case when running on a 4 core CPU computer and an average of 30 min when running with a 10 core CPU computer. The runtime varied per patient and was dependent on the size of the breast, which is associated with the size of the computational domain. In comparison with the outcomes provided by Gonzalez-Hernandez et al.^34^ on a 10 core machine running without parallel processing, the run time is halved from 1 h to 30 min. This shows how parallel processing has reduced the computation time and further improvements may be made by extending multithreading to the main detection algorithm, and using machines with larger number of cores.

**Table 2:** Error in tumor size from the IRI-Numerical Engine assessment compared to the actual from MRI.

Case	Predicted (mm)	Actual (mm)	Error (mm)	Absolute error (mm)
1R	12.9	14	− 1.1	1.1
2R	10	8	2.0	2.0
3R	10	9	1.0	1.0
4R	27.3	27	0.3	0.3
4L	10	8	2.0	2.0
5L	19.1	19	0.1	0.1
6L	18.9	17	1.9	1.9
7L	11.5	11	0.5	0.5
8L	10	12	− 2.0	2.0
9R	15	11	4.0	4.0
10R	10	11	− 1.0	1.0
11R	10	12	− 2.0	2.1
12L	10	5	5.0	5.0
13L	10	13	− 3.0	3.0
14R	10	5	5.0	5.0
15R	10	5	5.0	5.0
16R	27	26	1.0	1.0
17L	23.3	21	2.3	2.3
18L	10	5	5.0	5.0
19R	10	11	− 1.0	1.0
20L	10	9	1.0	1.0
21L	10	15	− 5.0	5.0
22L	10	5	5.0	5.0
23L	10	12	− 2.0	2.0

Negative values in tumor size correspond to the IRI-Numerical Engine predicting a smaller tumor diameter than actual.

## Discussion

The earlier study with a small sample of seven biopsy-proven breast cancer patients is expanded to a wider group of twenty-three patients with different breast sizes, tissue densities, and cancer types. The earlier study showed that the technique is very effective in detecting cancer (sample size of seven patients) and, encouraged by the success of this approach, the current study expanded to a larger number of patients. As the number of patients increases, the need for a more efficient image registration and detection algorithm was recognized and developed into the IRI-Numerical Engine. The IRI-Numerical Engine was able to accurately detect various types of breast cancers in various breast sizes and tissue densities. The twenty-three-patient study in this work showed the ability of the IRI-Numerical Engine to detect the presence and absence of breast cancer regardless of breast density, tumor grade, or cancer type. The results from Table 2 show that the largest absolute error in size occurs for small tumors for most of the cases (Case#: 12L, 14R, 15R, 18L, and 22L). The results also show that smaller tumors (< 1 cm) have equivalent metabolic heat generation as a 1 cm tumor. This is due to two factors: (i) growing tumors being more metabolic active and highly perfused in comparison to fully grown tumors, and (ii) the smallest tumor that can be modeled using Gautherie’s relation (Eq. 4) is 1 cm. The IR camera and algorithm is able to capture this high thermal distribution and interpret it as a 1 cm highly metabolically active and perfused tumor. Further study is needed in understanding the source of error, which may be related to tumor depth or position within the breast.

Conducting a breakdown of the patient cohort showed that IDC made up the majority of cancer types (75% of all cases) and SF tissue density made up the majority of breast density types (67% of all cases). Table 3 shows a full breakdown of the cancer type and breast density in the patient cohort. The two main types of noninvasive breast cancer are DCIS and LCIS, with DCIS being the most common and making up 16% of diagnosed cancer cases^43,44^. Although LCIS is a non-cancerous tumor, the condition is associated with abnormal growth in the breast lobules creating a high risk (12 times higher than average) of developing into invasive cancer^44,45^. The two main types of invasive cancers that are also the two most common breast cancers are IDC (80% of all cancers) and ILC (10% of all cancers)^43,44,46^. This shows that the patient cohort is a good representation of the overall breast cancer screening population. However, a larger clinical study is warranted on a statistically significant patient cohort size to determine the sensitivity and specificity of this method. To be able to establish a sensitivity of 90% with p value of 0.05 for the IRI-Numerical Engine, it is estimated that about 163 patients will need to be recruited for this clinical study. A similar estimation can be conducted to obtain patient size for the sensitivity, preferably around 90%. It was observed that most small tumor cases were IDC with one having ILC, one having LCIS, and one having ADH. The one case with ADH, case 12L, showed that the precancerous lesion had features closely resembling low grade DCIS as mentioned in the patient pathology report. Studies have shown the difficulty in distinguishing ADH from DCIS with some ADH cases being classified as borderline DCIS^47–49^. This shows the capability of the IRI-Numerical Engine to detect precancerous tumors. Furthermore, case 12L was one of the four HD cases showing that detection can be accomplished regardless of breast density. The percentage of breast densities in the patient cohort also paralleled the screening population. According to the CDC, the two most common breast types are SF (40% of all women) and HD (40% of all women) which are the two most common types in this work^50^.

**Table 3:** Breakdown of patient cohort in terms of cancer type and breast density. Percentage for largest cancer type and breast density type are shown in parentheses.

Cancer type		Breast density	
ADH	1/24	PF	3/24
DCIS	1/24	SF	16/24 (67%)
IDC	18/24 (75%)	HD	4/24
ILC	3/24	ED	1/24
LCIS	1/24		

In conclusion, the enhancements were conducted on the previous work on development of the IRI-Numerical Engine, which was able to correctly detect the presence and absence of breast cancer in seven breast cancer cases. These enhancements were mainly in the image registration process, ROI extraction process, the initialization process, and computational flow of the algorithm. The study was conducted on a total of twenty-three patients and it shows the capability of the IRI-Numerical Engine to detect breast cancer regardless of cancer type, breast density, and tumor grade. In this patient study, 75% of cases were IDC and the most common tissue types were SF and HD, which paralleled the breast cancer screening population. However, a more statistically significant clinical study with a larger patient cohort is needed to study the sensitivity and specificity of the algorithm. The results from this study showed that the mean, median, and maximum absolute error in tumor size prediction were 2.4 mm, 2 mm, and 5 mm, respectively. Tumors smaller than 1 cm had the largest error and were predicted as 1 cm due to being more metabolically active than larger tumors, as described by Gautherie^24^. This effect is quite small; however, further studies are needed to identify the size effects of the metabolic heat generation on the surface temperature. In order to make this technique clinically and commercially viable, further research is needed in generating digital models by alternative methods such as depth sensors, stereo vision cameras, and 3D scanners. Once the digital model is developed using these alternative methods, the IRI-Numerical Engine can be developed using open-source tools and software to implement in clinical environments. The results showed the potential of the IRI-Numerical Engine to detect earlier stage breast cancers with sizes smaller than 1 cm and precancerous tumors. Furthermore, the results showed that the breast density did not influence the detection of breast tumors, removing the masking effect associated in mammography screening. This helps set a path of having IRI as an inexpensive, accurate, and efficient adjunct to mammography that can help in reducing the recall rates and number of biopsies. This will also reduce the stress and anxiety women must endure from recalls and further procedures due to the masking effects of dense breast tissue.

## Methods

This work aims to further validate a novel IRI detection algorithm developed at Rochester Institute of Technology (RIT) using data from a clinical study conducted at Rochester General Hospital (RGH). The main research objective of the current work is to evaluate the efficacy of the IRI-Numerical Engine in a small sample size of twenty-three patients. A positive outcome, in terms of predictive ability, will be used to determine if the study should be expanded into a large sample set. From the results, it is clear that our methodology is accurately predicting the positive and negative cases, confirming the need to expand the study to a statistically meaningful larger sample size for determination of the sensitivity and specificity of this technique. The IRI-Numerical Method is an enhanced packaged version of the work conducted by Gonzalez-Hernandez et al.^34^ and Recinella et al.^41^ for a more efficient and accurate detection algorithm.

The main components of this study are as follows: (i) capturing of multi-view IR images in a clinical environment, (ii) generating patient-specific digital breast models (DBMs) from MRI for thermal modeling, (iii) conducting thermal simulations of breast cancer using ANSYS Fluent, and (iv) implementing a detection algorithm that utilizes the surface temperatures from multi-view IR images and thermal simulations to detect and predict the size and location of breast tumors. The IRI clinical imaging procedures and clinical data were first compiled by Owens^51^. The procedures for generating patient-specific DBMs, thermal simulation of breast cancer using the DBMs, and the detection algorithm were first developed by Gonzalez-Hernandez^52^. In this study, enhancements to the detection algorithm are conducted and packaged as the IRI-Numerical Engine. Details of each procedure, collected data, and enhancements are described below.

### Clinical setup and imaging

The clinical study started under an Institutional Review Board (IRB) protocol developed at RGH, where thirty biopsy-proven breast cancer patients were recruited. Pathology reports and other clinical data were collected from the thirty patients who were imaged with both MRI and IRI. Recruitment and imaging of patients was conducted between March 2018 and September 2019. Patients were recruited if they were reported to have a palpable breast mass, or a confirmed mass in the screening mammography. The patients that underwent biopsy for confirmation and pre-op MRI were selected for IRI image capture and analysis. Patients with missing MRI and IR data were not selected for this study, reducing the patient cohort from thirty to twenty-three. All patients were required to fill out an informed consent form, previously approved by the RGH IRB, to participate in the study. Human subjects training (Good Clinical Practice and Good Documentation Practice) and the Collaborative Institutional Training Initiative were conducted for all personnel working on the project. All patient data was de-identified before use in any of the methods described in this work. All methods in this work that utilized any patient data (MRI and IR images, and patient reports) followed the human subjects research guidelines and regulation established in the RGH IRB.

#### Image acquisition

**Figure 2:**
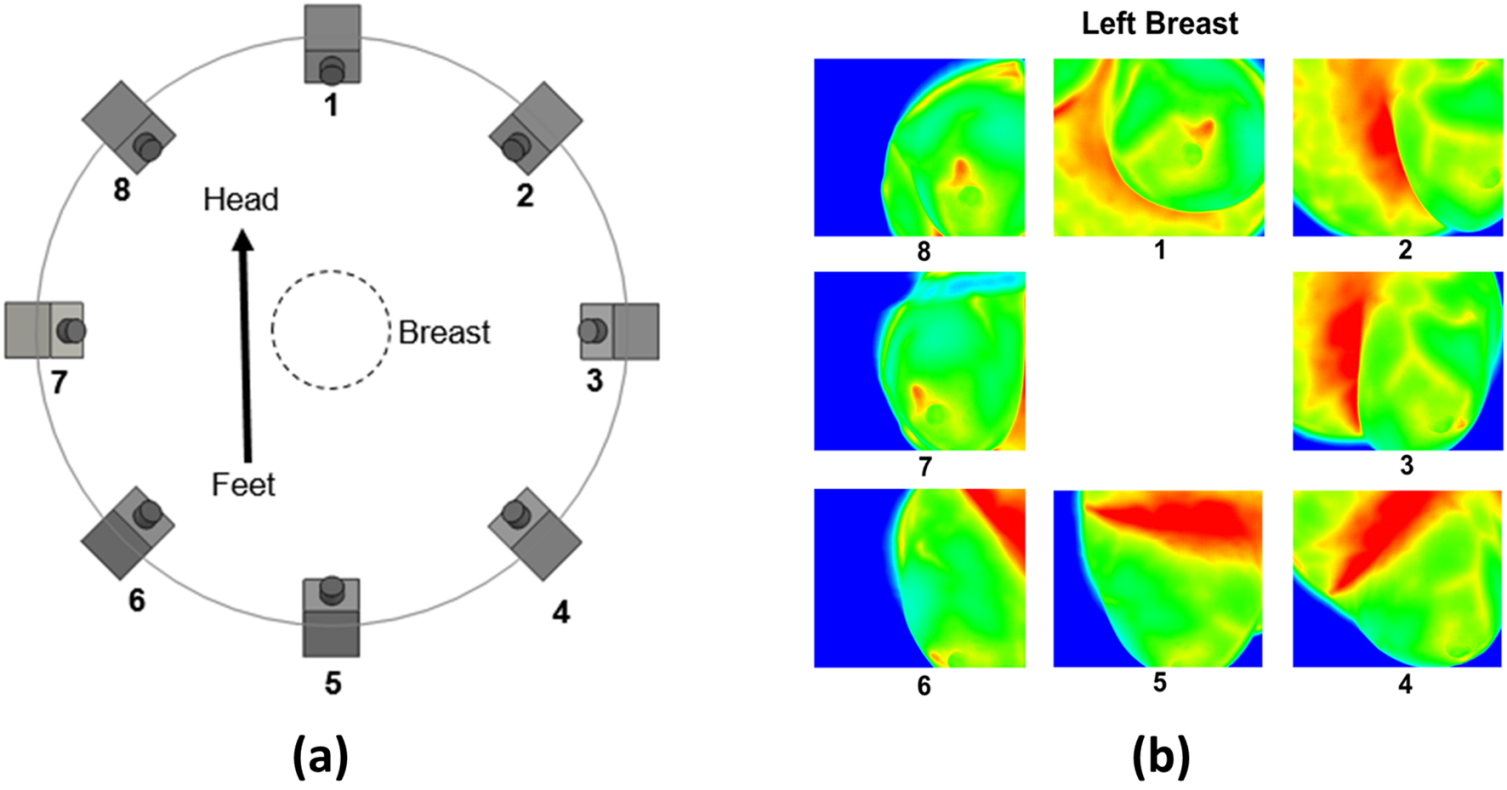
Depiction of IRI image capture setup with (**a**) illustrating the IR camera position relative to the body and (**b**) example IR images of a patient's left breast associated with the position number. All images are taken clockwise starting at the head of the patient with the camera tilted upwards at a tilt angle of 25° giving access to the chest wall in addition to the breast surface. Hot spots and breast vascular regions are captured in the IR images. The hotspot shows up in views 1, 7, and 8. Vascular regions are more prominent in views 1–4.

The MRI and multi-view IR images were both captured in the same prone position with a GE 3T MRI scanner and an inhouse IRI imaging system, respectively. The IRI imaging system consisted of a sturdy retrofitted imaging table with a 23 cm hole and an a FLIR SC6700 IR camera. This IR camera has a 640 × 512 pixel resolution and a thermal sensitivity of 20 mK, or 0.02 °C. The imaging table has a 5 cm layer of foam placed on top for comfort and a layer of disposable paper placed on top of the foam in accordance with hospital hygienic procedures. Patients were asked to disrobe from the waist up while wearing a hospital gown with an opening in the front and to lie down on the table in the prone position with one breast going through the hole. IR images were obtained at 8 views (45° intervals) at a 25° vertical tilt of each breast. Figure 2 shows an illustration of the IRI procedure and example IR images of a breast. IR images were captured after 10 min of acclimation to obtain steady-state temperatures of the breast surface. Details of image acquisition and setup, as well as patient recruitment and preparation are described in Recinella et al.^41^ and Owens^51^.

### Patient-specific thermal simulation of breast cancer

A method for generating patient-specific 3D breast geometries, or digital breast models (DBMs), from MRI data for thermal simulations of breast tumors was developed by Gonzalez-Hernandez^52^. The method utilized image processing and 3D reconstruction to generate the models. This work utilizes this method to generate patient-specific DBMs from MRI for the additional patients. The MRI data was only used to generate the computational domain for thermal analysis, but not to model the tumor shape, size, or position. Any tumor information from the MRI or other clinical data was strictly used for comparison and validation purposes.

#### Model generation

The patient-specific DBM generation method established by Gonzalez-Hernandez et al.^33^ was utilized to generate models for sixteen additional patients using the available MRI data, giving thirty-two additional DBMs for analysis. The method consists of image processing methods such as noise filtering, edge detection and segmentation, as well as 3D reconstruction and computer graphic methods. The steps for patient-specific DBM generation are as follows:

1. Select the breast and a small region that connects to the chest wall.
2. Filter any noise in the MRI using a median filter on each MRI slice.
3. Conduct edge detection to outline the breast shape for each MRI slice.
4. Use the outlines to segment the entire breast shape on each MRI slice.
5. Generate a 3D geometry by combining the segmented MRI slices.
6. Conduct 3D smoothening of the breast model.
7. Separate the breast model into a left breast model and a right breast model.

**Figure 3:**
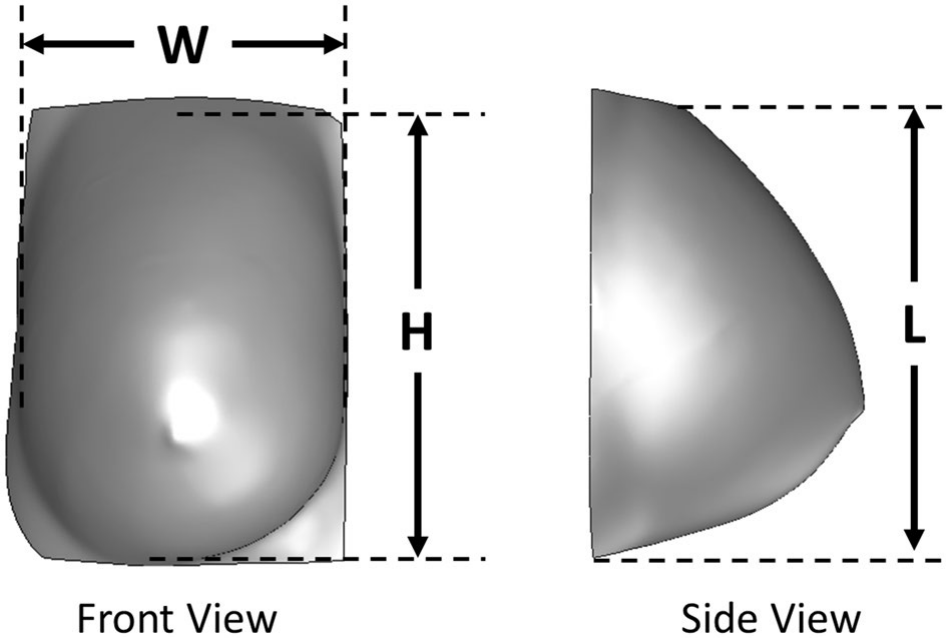
Example frontal and side view of Patient 11 DBM utilized for obtaining the height (H), width (W), and length (L), also known as the geometric characteristic measurements. These values are measured at the point of contact between the breast and the chest wall.

More details in relation to these steps and model generation are provided in Gonzalez-Hernandez et al.^33^ It is important to note that model generation is not limited to 3D reconstruction from MRI and can be conducted utilizing any other methods that can obtain the breast shape, such as a 3D scanner. The MRI is not needed in this technique: it was used for generating digital models since it was available. The digital model is then used for inverse heat transfer analysis in detecting tumors. Geometric characterization of the models was conducted by utilizing the geometric parameters W, H, and L^34,41^, as shown in Fig. 3. In Fig. 3, W and H are the width and height of the breast, respectively, measured from the front view in the x- and z-direction, respectively, and L is the length of the breast measured from the side view in the y-direction. A list of these measurements for each patient with their respective digital breast model is shown in Table 4.

**Table 4:** The geometric characteristic measurements of the generated patients-specific DBMs for all twenty-three patients.

Patient	Right breast			Left breast		
	H (cm)	W (cm)	L (cm)	H (cm)	W (cm)	L (cm)
1	8.82	5.69	5.19	8.96	5.51	5.06
2	9.10	6.00	10.38	9.10	5.95	10.19
3	10.24	7.25	10.13	10.10	6.88	10.45
4	9.66	11.65	9.01	9.52	11.71	9.40
5	12.60	11.82	12.53	12.88	11.41	12.51
6	10.06	11.06	12.02	10.06	10.73	11.73
7	11.34	4.78	7.10	10.92	5.27	6.26
8	14.89	14.36	10.03	16.41	16.15	10.34
9	13.97	10.04	7.08	13.57	10.37	7.01
10	13.49	13.25	5.18	10.84	12.88	5.26
11	15.14	14.26	10.78	15.00	14.45	11.37
12	12.81	8.81	9.15	14.33	9.03	7.20
13	11.74	13.32	7.36	12.50	11.89	4.66
14	10.46	8.27	6.68	10.43	9.12	7.86
15	14.80	13.53	10.82	13.89	12.76	11.19
16	17.04	15.72	11.64	16.87	14.96	11.17
17	12.16	10.89	7.79	10.85	10.29	8.83
18	11.98	7.81	6.26	12.28	8.24	7.54
19	14.19	8.05	6.10	15.96	8.65	7.62
20	8.76	8.79	7.35	8.18	8.98	7.84
21	14.57	9.78	10.39	11.55	9.86	10.51
22	15.58	9.08	6.87	14.97	9.07	6.49
23	11.16	13.04	9.34	10.40	14.93	9.26

#### Terminology

The terminology of key words utilized throughout this work are as follows:

- *Breast Tumor* a mass of abnormally grown breast tissue.
- *Tissue Thermal Properties* breast tissue thermal characteristic properties, such as thermal conductivity and blood perfusion, which determine how heat is transferred within the tissue.
- *Tumor Characteristics* the major characteristics of the tumor that affect the heat distribution on the surface of the breast captured by the IR images. The major characteristics include the tumor’s metabolic heat generation rate and blood perfusion rate. The metabolic heat generation rate is a consequence of the increase in the tumor’s cellular metabolism, while the blood perfusion rate is a consequence of the increased vasculature of the tumor due to angiogenesis.
- *Bioheat Transfer Equations* heat transfer equations utilized to model and describe the temperature distribution accounting for the heat transfer effects of biological entities inside the breast. In this work, Pennes's bioheat equation is utilized, which accounts for the heat conduction in the tissue, metabolic activity of the tissue, and convective heat transfer from blood perfusion.
- *Boundary Conditions* the thermal conditions of the environment interacting with the breast at specified boundary areas. In this work, the boundary conditions include the temperature at the chest wall, ambient temperature interacting at the breast surface, and the convection heat transfer due to natural convection from the ambient environment.
- *Thermal Simulations* numerical methods, such as computational fluid dynamics (CFD) or finite difference methods, which are utilized to solve for the temperature distribution from heat transfer equations in complex problems or geometries through discretization of the geometry. In this work, ANSYS Fluent CFD software is utilized to discretize the geometry and solve the temperature distribution of Pennes’s bioheat equation.
- *Inverse Heat Transfer* inverse modeling algorithms for heat transfer equations that utilize an iterative approach to solve for unknown parameters using known temperature distributions or temperature data at specified points. In this work, the Levenberg–Marquardt algorithm^53^ method is utilized with surface temperatures from patient IRI data to find the tumor heat source size (tumor diameter) within the breast geometry. The tumor heat source is placed outside the breast geometry when there is no tumor present.
- *Model Validation* refers to comparing the predictive value with the MRI images for tumor size and pathological data for the cancer type.

#### Bioheat transfer modeling

Thermal simulations of breast cancer were conducted using ANSYS Fluent, Pennes’s bioheat equation^25^ and the findings by Gautherie^24^. In this work, tumors were modeled as metabolically active and highly perfused heat sources located within the breast. For thermal modeling, the healthy tissue and cancerous tissue region were modeled using the following:

2 $$\nabla \cdot \left( {k_{h} \nabla T} \right) + \rho_{b} c_{b} \omega_{h} \left( {T_{a} - T} \right) + Q_{h} = 0$$

3 $$\nabla \cdot \left( {k_{t} \nabla T} \right) + \rho_{b} c_{b} \omega_{t} \left( {T_{a} - T} \right) + Q_{t} = 0$$

4 $$Q_{t} = \frac{{3.27 \times 10^{6} }}{{468.5\ln \left( {100d_{t} } \right) + 50}}$$

5 $$k_{h} \left. {\frac{\partial T}{{\partial {\varvec{n}}}}} \right|_{A,B,C,D} = 0$$

6 $$T_{E} = T_{body}$$

7 $$\left. { - k_{h} \frac{\partial T}{{\partial {\varvec{n}}}}} \right|_{F} = h\left( {T - T_{\infty } } \right)$$

where the subscripts $h$ and $t$ describe the healthy and cancerous, or tumor, regions, respectively. These heat sources are implemented in ANSYS Fluent through a user-defined function (UDF). Once the DBMs were generated, thermal simulations using ANSYS Fluent software were conducted using a steady-state Pennes’s bioheat equation^25^ for healthy tissue (Eq. 2) and tumor tissue (Eq. 3) with metabolic activity of the tumor (Eq. 4) from the work established by Gautherie^24^. A convective boundary condition is assigned to the breast surface (Eq. 5 and label F), constant temperature is assigned to the chest wall (Eq. 6 and label E), and no heat flux boundary conditions are assigned to the side and top faces of the model (Eq. 7 and labels A–D). Computed temperatures images, also known as computed images, are generated after every thermal simulation involving the DBMs.

The healthy tissue region (Eq. 2) consists of a metabolic heat generation source term $Q_{h}$ and a perfusion heat sink term $\rho_{b} c_{b} \omega_{h} \left( {T_{a} - T} \right)$. The perfusion heat sink term models the body’s regulatory system due to blood flow in the local vasculature regions. This simplified model has been utilized throughout literature to accurately represent the regulatory system while reducing the need to utilize more complex vasculature thermal models^54^. The cancerous tissue region (Eq. 3) consists of a metabolic heat generation term of the tumor $Q_{t}$ obtained from Eq. 4, which relates the metabolic heat generation as a function of the tumor diameter^24^. In addition, the effects of tumor angiogenesis and the tumor microenvironment are modeled through a perfusion heat source $\rho_{b} c_{b} \omega_{t} \left( {T_{a} - T} \right)$. Both the metabolic heat generation term and perfusion heat source are based on the findings of Gautherie^24^ on malignant tumors being highly perfused and metabolically active.

**Figure 4:**
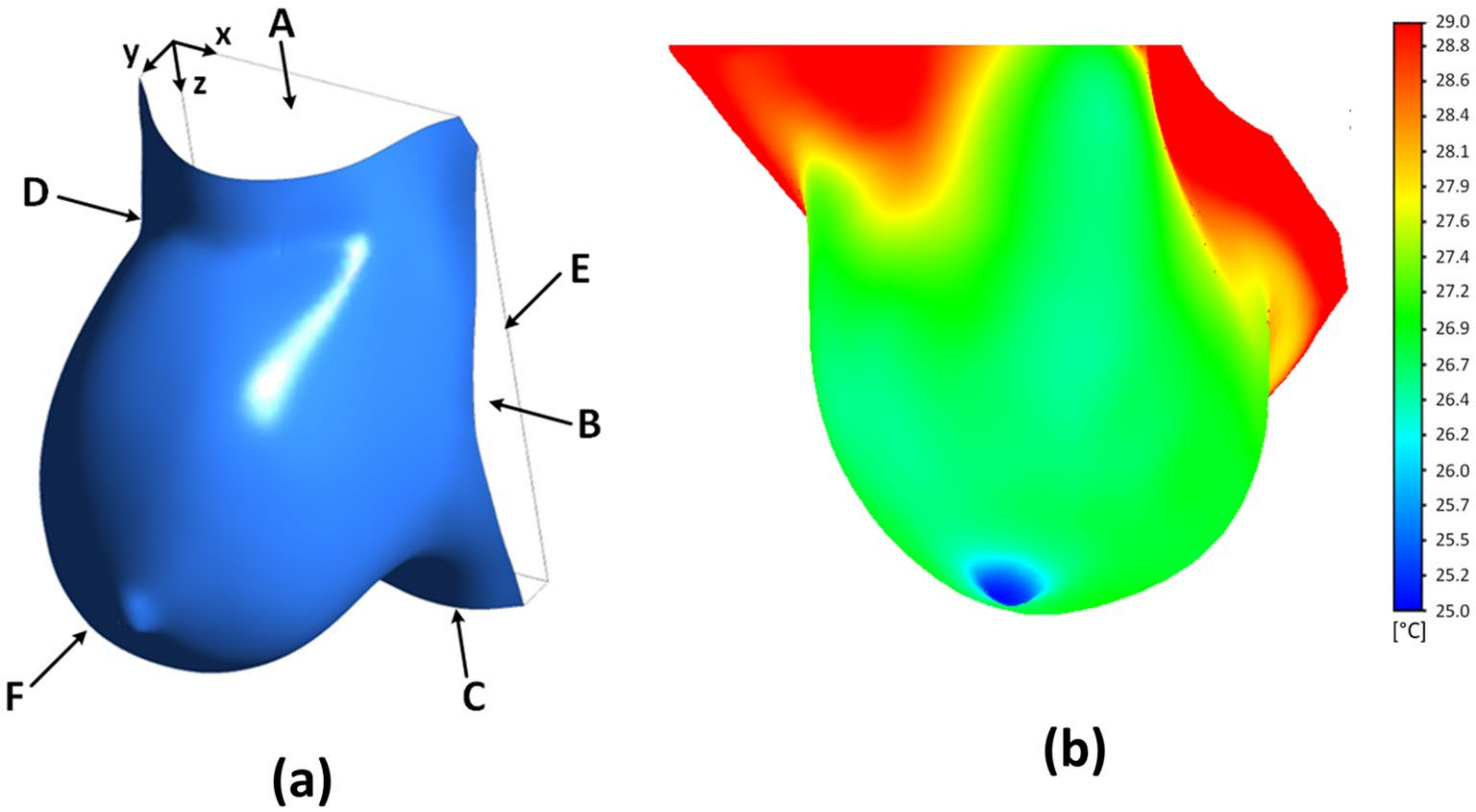
Example of a (**a**) patient-specific DBM with labeled boundary regions and (**b**) thermal simulation of breast cancer for the same patient-specific DBM. Labels A–D are the side regions that are given a no heat flux boundary condition. Label E is the chest wall region given a constant temperature boundary condition. Label F is the breast surface region given a convection boundary condition.

The boundary conditions utilized in this method, Eqs. 5–7, were implemented on the model shown in Fig. 4a. The thermal simulation shown in Fig. 4b depicts a temperature distribution on the breast surface resulting from a tumor as a heat source based on its metabolic activity and the blood perfusion rate. The thermal physical property values utilized for thermal simulation of breast cancer in the work developed by Gonzalez-Hernandez^52^ and further validated by Gonzalez-Hernandez et al.^33,34^ are shown in Table 5. The values from Table 5 are utilized in every digital breast model for this study. Previous studies have shown that the metabolic activity and location of the tumor played a significant role in the surface temperature while the other properties were negligible^28,30,34,54^. Gonzalez-Hernandez et al.^34^ conducted a thermal sensitivity analysis to validate that the tumor size and locations have an effect on the detection of breast tumors. This was due to the relationship between the metabolic activity and the tumor diameter based on the findings from Gautherie^24^ represented in Eq. 4. More details of the modeling and thermal simulation process is described in Gonzalez-Hernandez^52^ and Gonzalez-Hernandez et al.^33^.

**Table 5:** Table of thermal physical properties utilized for thermal simulations of breast cancer obtained from literature as provided by Gonzalez-Hernandez et al.^33,34^.

Properties	Value
Perfusion rate of health tissue ($\omega_{h}$)	$1.8 \times 10^{ - 4} {\text{ s}}^{ - 1}$
Perfusion rate of tumor ($\omega_{t}$)	$9 \times 10^{ - 3} {\text{ s}}^{ - 1}$
Metabolic activity of healthy tissue ($Q_{h}$)	$450{\text{ W m}}^{ - 3}$
Temperature of arteries ($T_{a}$)	$310{\text{ K}}$
Specific heat of blood ($c_{b}$)	$3840{\text{ J kg}}^{ - 1} {\text{ K}}^{ - 1}$
Density of blood ($\rho_{b}$)	$1060{\text{ kg m}}^{ - 3}$
Heat transfer coefficient ($h$)	$13.5{\text{ W m}}^{ - 2} {\text{ K}}^{ - 1}$
Core Temperature ($T_{body}$)	$310{\text{ K}}$
Ambient temperature ($T_{\infty }$)	$294{\text{ K}}$

### IRI-numerical engine

Using the methods to generate patient-specific DBMs and conduct thermal simulations of breast cancer, an inverse heat transfer approach was developed with IR images to detect and localize a tumor^34,41,51,52,55^. This approach utilized the surface temperatures of the breast captured by the IR camera and compared them to the surface temperatures of the thermal simulated model. This algorithm then tries to back calculate the tumor size and location to match the surface temperatures of the IR images. In the absence of a tumor, the algorithm places the tumor either along the chest boundary wall or the farthest outside corner of the domain to simulate the lack of heat source. This is due to an additional constraint to the algorithm that places the tumor within the computational domain. When the algorithm places the tumor at the boundary and corner points, the effect of this tumor heat source is negligible. This allows the algorithm to be utilized to detect the presence and absence of a tumor based on the presence or absence of a heat source in the breast domain and its effect on the surface temperature. Recinella et al.^41^ showed that the temperature from local vasculature captured by the IR camera did not have an effect on the detection of breast cancer. This is due to the larger effect of the tumor heat source in comparison to the localized thermal variations from the breast’s vasculature. Enhancements to this algorithm were conducted for this work in the form of an enhanced image registration, improved computational flow, and parallel processing for thermal simulation.

#### Inverse heat transfer approach

**Figure 5:**
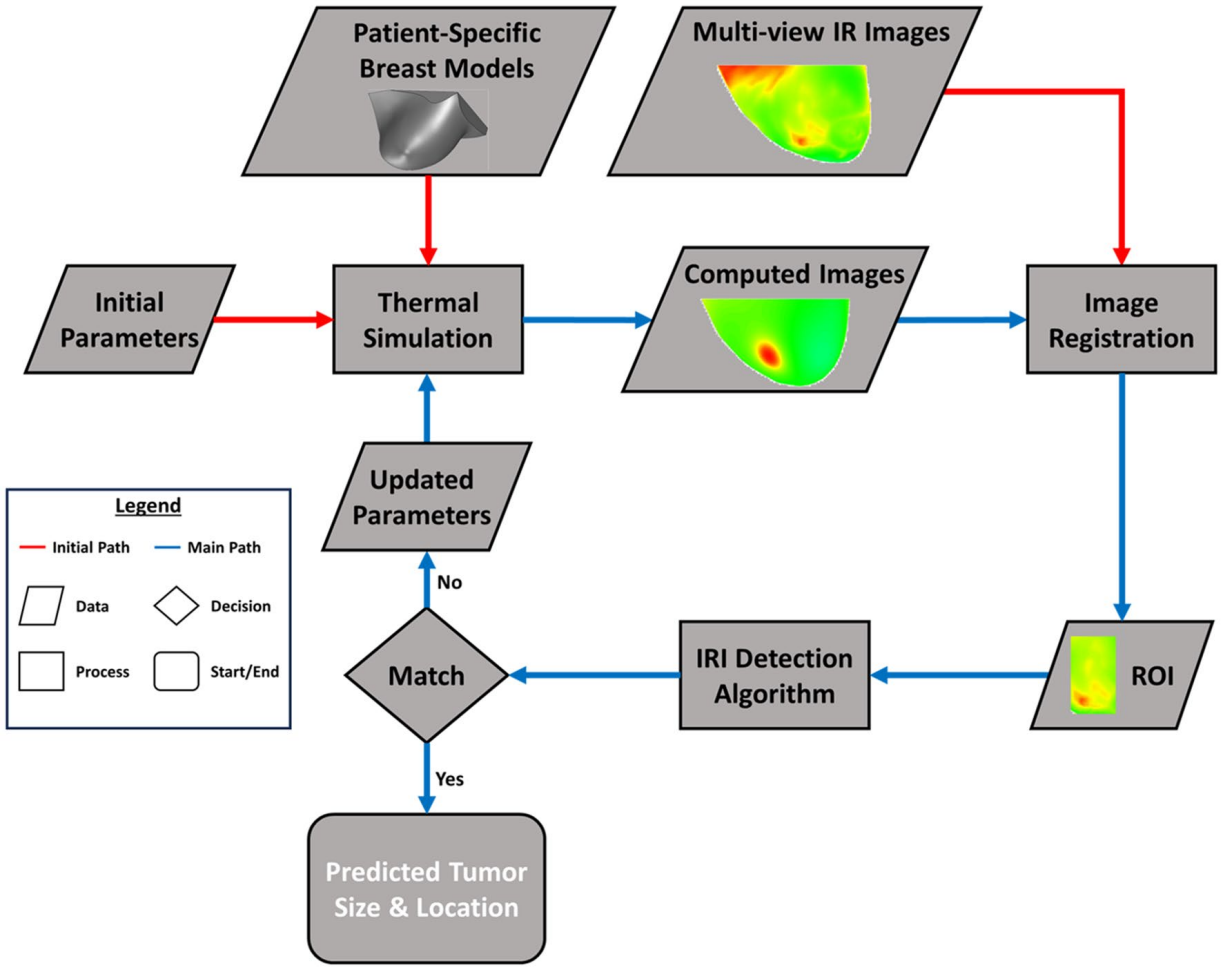
Flowchart of the inverse heat transfer breast detection process using IR images developed by Gonzalez-Hernandez^52^ and validated by Gonzalez-Hernandez et al.^34^. The process for the algorithm starts with the thermal simulation conducted on the patient-specific model with initial parameters. This generates a computed image that gets aligned, or registered, with the IR image at the corresponding view. The ROI is extracted for the given views and are analyzed by the IRI detection algorithm. If there is no match, a new parameter is generated for thermal simulation and the cycle starts all over again. If there is a match, then the algorithm ends, and the outcome is given. Any red arrow indicates the initial inputs needed for the algorithm to start. The main detection process can be followed using the blue arrows. The flowchart legend on the left shows this distinction between paths as well as the distinction and significance of the shapes. A rhombus indicates the typing to be a data. A diamond indicates a decision process. A rectangle indicates a computational or algorithm process. And a rounded rectangle indicates the outcome of the algorithm.

In the inverse heat transfer approach, computed temperature images of the simulated model were generated from the thermal simulation and compared with corresponding IR images through image registration and an iterative inverse heat transfer algorithm known as the Levenberg–Marquardt algorithm^53^. Figure 5 shows the flowchart for this developed algorithm replicated from Gonzalez-Hernandez et al.^34^. This algorithm was validated with seven biopsy-proven breast cancer patients and is a preliminary basis to this work. An initial tumor of size 1.8 cm was placed at the center of each breast model and simulated to obtain computed images. This is conducted for any case regardless of the presence or absence of a breast tumor. Gonzalez-Hernandez et al.^34^ showed that the final outcomes of the algorithm were independent of the initial guess. This work utilizes this initial guess as a standardization method for all patient cases. Image registration was conducted on the computed and IR images where a Region of Interest (ROI) for each was obtained and analyzed by the inverse heat transfer algorithm, also known as the IRI detection algorithm. Gonzalez-Hernandez et al.^34^, through a sensitivity analysis, established that the size of the ROI played a role in computational time but had minimal effect on the detection results. The algorithm checks to see if the surface temperatures in the ROIs match. If they do not match, the algorithm generates new parameters for the thermal simulation to generate a new computed image based on the new tumor size and location. This was conducted until the algorithm was able to predict the tumor size and location corresponding to the temperatures of the IR images. Further details of the IRI detection algorithm and process are described in Gonzalez-Hernandez^52^ and Gonzalez-Hernandez et al.^34^.

#### Enhanced image registration

**Figure 6:**
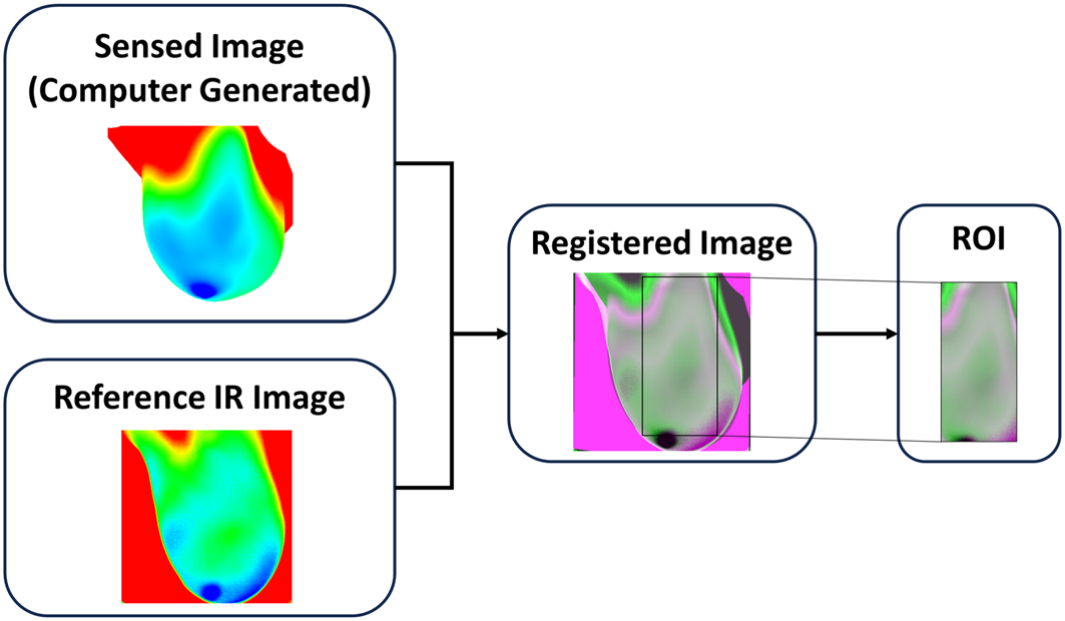
Example of the enhanced image registration and ROI extraction processes utilized in this work. Image registration is conducted between the computer-generated image (sensed image) and the reference IR image. A registered overlap image is then created and utilized to obtain the ROI that will be utilized by the detection algorithm.

The IR images and MRI images of the patients were both captured in the prone position, making image registration an effective technique to align the surface temperatures between the IR images and DBM. Image registration is the process of aligning two or more images by geometrically transforming the images to match the spatial coordinates of another image called the reference image^56^. The images that are being transformed to match the reference image are called the sensed images. In this work, the referenced images are the IR images, also referred to as the referenced IR images, and the sensed images are the computed images generated from the thermal simulations. The study conducted by Gonzalez-Hernandez et al.^34^ utilized an intensity-based multimodal similarity image registration algorithm using MATLAB’s Image Processing Toolbox. In this study, an intensity-based multimodal affine image registration algorithm was utilized on the IR reference images and sensed images. Prior to image registration, pre-processing was conducted by converting all images from RGB to grayscale as required by the MATLAB image registration function^57^. After pre-processing, the optimizer and metric for the image registration function had to be configured to change the registration type to multimodal image registration^58^. The transformation type was selected to be an affine transformation, which allows the sensed image to translate, rotate, scale, and shear so that it matches the reference images. The intensity-based multimodal affine image registration enhanced and aided the registration process as well as accounted for any distortion captured by the IR camera. This enhanced image registration process was packaged into an algorithm that allows user interaction through two main user-interfaces (UIs). In the first UI, the user can select the portion of the image containing the breast to go through the image registration process. Once the user has selected the breast area, the ROI can be selected for analysis through the second UI. Figure 6 shows an example of the image registration process and example outcomes provided by the enhanced algorithm.

#### Improved computational flow

**Figure 7:**
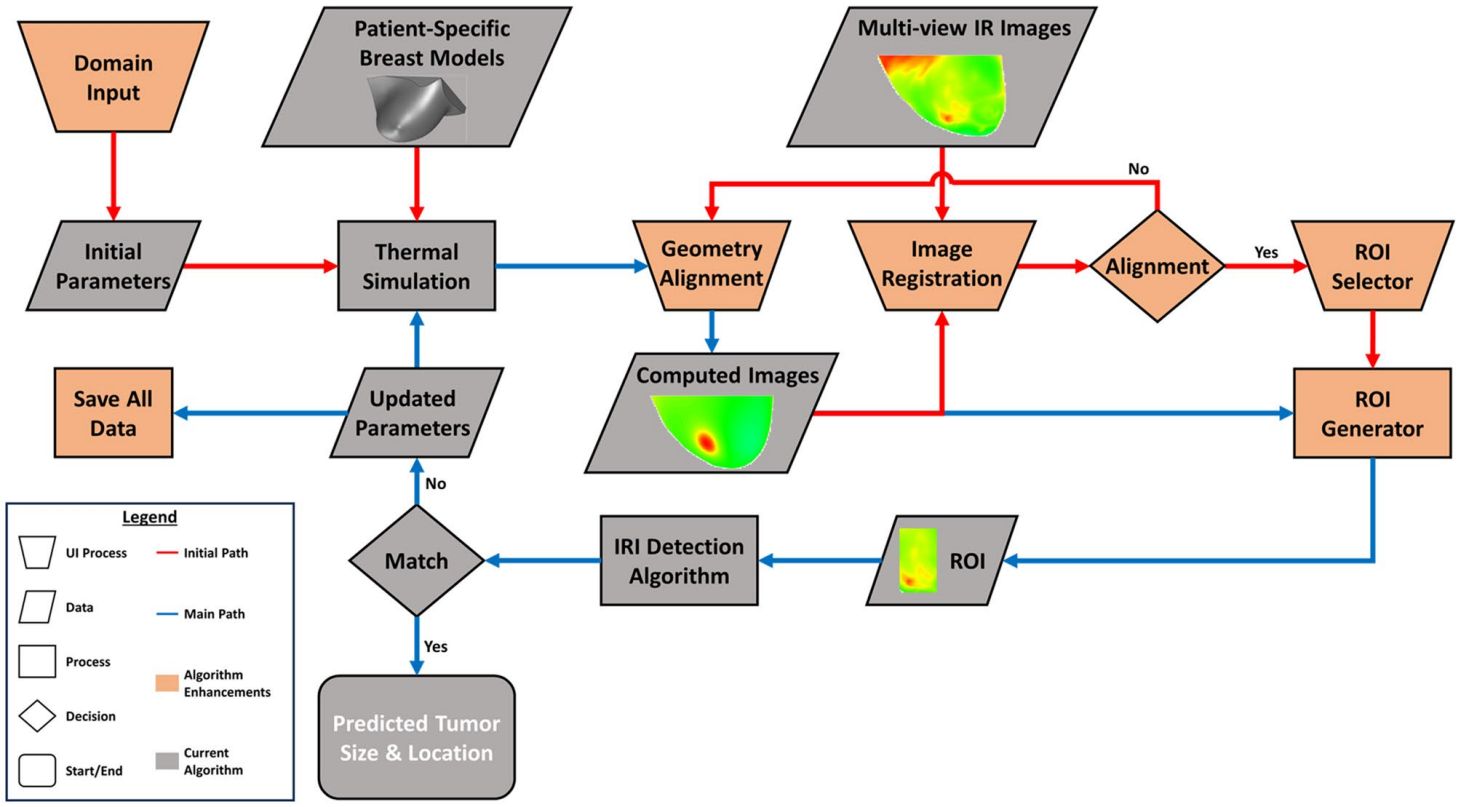
Flowchart for the IRI-Numerical Engine for enhanced and robust breast cancer detection. The process starts with the IRI-Numerical Engine asking the user through the Domina Input UI to manual input the domain of the patient-specific model to generate the initial parameters. The patient-specific model and initial parameters are then utilized by ANSYS Fluent to conduct thermal simulation of breast cancer. The Geometric Alignment UI in Fluent then rotates and moves the model around to match the IR image views and once finished computed images are generated. The computed images and associated IR images go through an Image Registration UI which logs the images and compares them through an alignment decision algorithm. If they are not aligned, the Image Registration UI waits for new computed images for comparison. If they are aligned, the ROI Selector UI is activated, and the user can select the ROI to be generated by the ROI Generator. From here the process is the same as the inverse heat transfer breast detection process shown in Fig. 5, but a fail-safe procedure is conducted by saving after the creation of the new parameters. The flowchart legend on the left shows the significance and distinction between shapes, shape colors, and arrow colors. All enhancements are shown in orange and the one new shape added is the trapezoid which indicates a UI process.

To improve workflow and computational flow of the IRI-Numerical Engine, enhancements to the algorithm are developed. The enhancements include coupling the image registration algorithm with the detection algorithm, UIs, and the development of fail-safe procedures. Figure 7 shows the flowchart for the IRI-Numerical Engine algorithm with the enhancements conducted in this work. One of the first enhancements that was added is a Domain Input UI which allows the user to input the computational domain of the patient-specific model. The computational domain values can be obtained directly from ANSYS Fluent to have more precise spatial coordinates for the tumor location. This UI is utilized to generate the initial tumor based on the computational domain of the DBMs. Another enhancement added to the algorithm is the coupling of the enhanced image registration process and the detection algorithm. Previously, manual image registration was conducted and tested prior to running the IRI-Numerical Engine to ensure alignment of the sensed and IR images. In this work, the image registration setup process is part of the algorithm through several UIs incorporated with the image registration algorithm described in the previous section. The purpose of these UIs is to ensure the registration and ROI extraction were conducted correctly by checking with the user and allowing the user to restart or reselect when needed. If the user selects to restart, geometry re-alignment is conducted in ANSYS Fluent where the camera is rotated to the appropriate view and new computed images are saved. One main feature of the IRI-Numerical Engine is a fail-safe procedure that ensures that all data and setups generated by the IRI-Numerical Engine were saved at every iteration. This allows for the analysis to continue from the previously saved iteration even after any type of malfunction.

#### Incorporation of parallel processing

Another main feature of the new algorithm was the incorporation of parallel processing of ANSYS Fluent thermal simulation for faster computational time. Parallel processing is the computational method that allows computational processing to be split and simultaneously computed through multiple threads of a computer processing unit (CPU) or graphics processing unit (GPU), multiple CPUs or GPUs, or a cluster of computers^59^. In ANSYS Fluent, parallel processing is conducted by partitioning the computational mesh and assigning each partition to a compute node to be processed simultaneously^60^. For this work, the simulations and IRI-Numerical Engine were performed on two machines: (i) Intel^®^ Core™ i7-6700 3.40 GHz workstation with 4 cores, 8 threads and 32 GB RAM, and (ii) Intel^®^ Xeon^®^ E5-2630 v4 2.20 GHz with 10 cores, 20 threads and 32 GB RAM. Parallel processing was set up using the ANSYS Fluent UI with simulations utilizing all threads for each machine. All UDFs that were utilized in ANSYS Fluent to incorporate Eqs. 2–4. To read the tumor parameters, the UDFs had to be rewritten to run in parallel processing. The UDFs were further enhanced by incorporating functions that interacted with the main IRI detection algorithm and the fail-safe procedures.

## Data Availability

The datasets generated during and/or analyzed during the current study are not publicly available due to the confidentiality of patients but are available from the corresponding author on a reasonable request.
